# Prevalence, patterns of cytopenia, and its associated risk factors among HIV-positive adults on HAART at ART follow-up clinics in Dawuro Zone public hospitals, Dawuro, Ethiopia, 2025. Multi-center-based cross-sectional study

**DOI:** 10.1371/journal.pone.0358743

**Published:** 2026-11-13

**Authors:** Adimasu Abate, Segni Gemechu Hurisa, Mohammed Ibrahim, Shimelis Mitiku

**Affiliations:** 1 Department of Medical Physiology, College of Health Science, Wolaita Sodo University, Wolaita Sodo, Ethiopia; 2 Department of Medical Physiology, School of Medicine, Ambo University, College of Health Science and Referral Hospital, Ambo, Ethiopia; 3 Department of Biomedical Sciences, Institute of Health, Jimma University Medical Center, Jimma, Ethiopia; Ankara University: Ankara Universitesi, TÜRKIYE

## Abstract

**Background:**

*Cytopenia,* defined as a critical reduction in one or more hematopoietic lineages, remains the most prevalent immuno-hematological complication among HIV-infected individuals. Its multifactorial etiology involves direct viral suppression of the bone marrow, secondary opportunistic infections, and the adverse effects of Highly Active Antiretroviral Therapy (HAART). Clinically, cytopenias are associated with accelerated disease progression, suboptimal therapeutic response, and increased morbidity. Despite these significant health implications, there is a paucity of data regarding the prevalence, patterns, and associated risk factors of cytopenia among HIV-positive adults on HAART in the Dawuro Zone of Ethiopia.

**Objective:**

*To* assess the prevalence, patterns, and associated risk factors of cytopenia among HIV-positive adults on HAART in ART follow-up clinics in the Dawuro Zone public hospitals.

**Method:**

*A* multi-center, cross-sectional study was conducted from March 1 to April 30, 2025, across three public hospitals in the Dawuro Zone [i.e., Tercha General Hospital, Tocha Primary Hospital, and Gessa Primary Hospital], Ethiopia. Eligible participants were recruited from HIV patients on HAART at the follow-up clinics who were randomly selected until the target sample size of 231 was achieved. The study aimed to determine the prevalence and pattern of cytopenia using plain and ethylene diamine tetra-acetic acid (EDTA) for CBC analysis as per the WHO definition of hematologic abnormalities. In addition, cytopenia-associated risk factors like sociodemographic, clinical, nutritional, medication, behavioral factors, comorbidities, and the presence of opportunistic infections were assessed via interviewer-administered structured questionnaires. Univariate analyses were performed to establish the prevalence of cytopenia. Potential risk factors were examined using bivariate logistic regression at a 95% confidence interval (p < 0.25), with significant variables subsequently entered into a multivariate binary logistic regression model for statistical adjustment using SPSS v27, intending for statistical adjustment.

**Result:**

*The* study included 231 participants with response rates of 230 (99.6%). The prevalence of cytopenia was 64 (27.8%), of which 34 (14.8%) were unicytopenia, 23 (10%) had bi-cytopenia, and only 7 (3%) had pancytopenia. In multivariate analysis, cytopenia was significantly associated with age > 50 years (AOR = 5.1, 95% CI: 1.3–2.1, P = .023), male gender (AOR = 2.8, 95% CI: 1.2–6.5, P = .014), bedridden patients (AOR = 6.0, 95% CI: 1.31–27.2, P = .02), having opportunistic infection (AOR = 4.57, 95% CI: 1.1–18.4, P = .032), LPV/r-based ART regimen (AOR = 6.06, 95% CI: 1.1–26.6, P = 0.02), poor drug adherence (AOR = 6.97, 95% CI: 2.9–16.6, P = 0.0), and participants with a CD4 count < 200 cells/mm^3^ and 200–499 cells/mm^3^ at AOR = 3.5 and 3.4; *p* = 0.04 and 0.023, respectively.

**Conclusion and recommendation:**

In this study, the prevalence of cytopenia among adult HIV-positive adults was 27.8%, indicating a moderate burden. Male gender, older age, bedridden patients, opportunistic infections, LPV/r-based regimens, poor drug adherence, and CD4 counts below 499 cells/mm^3^ were significant associations with cytopenia. The routine and frequent CBC tests, and adequate adherence counseling before HAART and during follow-up time, may improve cytopenia among HIV-positive adults.

## Introduction

The human immunodeficiency virus (HIV) has emerged as a significant global public health issue. Currently, approximately 39.9 million people are living with HIV, and 630,000 people died from HIV-related cases worldwide in the year 2023 [1]. Among the affected regions by the pandemic, Africa has one of the highest rates of infection, with an estimated 26.0 million people living with HIV and 390,000 deaths attributed to HIV-related causes in 2023 [1].

Human Immunodeficiency Virus (HIV) infection continues to be a major public health issue, resulting in many immunohematological complications. Cytopenia is a major cause of morbidity and mortality, according to the World Health Organization, and it poses a financial burden with an estimated $47.8 billion spent on healthcare each year globally [1]. Among these, the most common blood complication among HIV-positive individuals, with a prevalence rate that varies from 1.35% to 95% experiencing Anemia [2], 4% to 40% experiencing Thrombocytopenia [3] worldwide.

Following transmission and entering cells, HIV rapidly replicates within the host and triggers an immune response [4]. This response is marked by the release of pro-inflammatory cytokines and chemokines, activation of polyclonal B-cells, and a gradual decrease in CD4 + T-cells. The virus can also bind to CD4-negative blood cells, such as erythrocytes, in the presence of complement receptor type 1 (CR1), also known as the C3b/C4b receptor or CD35. This ongoing immune activation and inflammation play a role in the disease progression and outcomes for those infected with the virus [4]. Although the clinical effects of HIV infection vary widely, various hematologic issues, particularly cytopenia (anemia, leukopenia, neutropenia, and thrombocytopenia), are often among the first and most common complications, occurring regardless of other symptoms [5,6].

Cytopenia can arise from various factors, including inflammation, infections, malnutrition, cancer, and the use of multiple medications [7]. The causes may be either the direct effects of HIV or its indirect influence on blood cell production. Direct effects involve the impact of HIV on hematopoietic stem cells (HSCs)/progenitor cells, while indirect effects can occur independently of HIV/HSPC interactions, such as those induced by drugs or co-infections, as well as the virus’s influence on cytokine signaling affecting HSCs [8]. Numerous adverse effects, including the onset of AIDS, are associated with cytopenia in individuals living with HIV, particularly anemia. Increased mortality rates, primarily linked to anemia, lead to more hospitalizations as well as a heightened risk of secondary infections, such as fungal and bacterial infections related to neutropenia [9,10].

In developed countries, the situation with HIV-related cytopenia is significantly different as compared to developing countries. These are due to advanced monitoring capabilities, well-established healthcare systems, and a variety of treatment alternatives, which are all advantages of these places. Research findings suggest that 65% of HIV-positive individuals develop some form of cytopenia; nevertheless, the availability of sophisticated treatment options and regular monitoring has significantly improved patient outcomes and severity of findings associated with the progression of AIDS and suppression of CD4 T cells [11,12]. However, the situation in developing nations is more difficult to understand. The study’s findings suggest that up to 84% of infected people have HIV-associated cytopenia, which is far more common in resource-constrained environments, especially in Sub-Saharan Africa, Southeast Asia, and portions of Latin America, compared to industrialized countries.

Anemia, the most prevalent hematological disorder in HIV-infected patients, arises from multiple mechanisms that impair the survival and function of hematopoietic stem progenitor cells in the bone marrow [8]. Cytokine deregulation can affect hematologic progenitor cells, with elevated Interleukin-6 (IL-6) increasing hepcidin levels, leading to iron retention in macrophages and reduced blood iron, which hampers hemoglobin production [7]. Additionally, cytokines like Interleukin-1β (IL-1β) and tumor necrosis factor (TNF-α) inhibit erythropoietin production, affecting erythrocyte precursor proliferation [13]. Deregulated hepcidin also degrades ferroproteins, reducing dietary iron absorption and iron mobilization from old red blood cells, further impacting hematopoiesis [14].

Platelets are tiny blood cells crucial for blood clotting, clot formation, and the healing of wounds. Thrombocytopenia, or low platelet count, is frequently observed in people infected with HIV, typically ranging from mild to moderate. However, there are instances of severe platelet reduction, dropping below 50,000/μL(15). Nowadays, with the use of combination antiretroviral therapy, this condition is more often seen in patients with uncontrolled HIV replication and co-infection with hepatitis C [15]. The mechanisms behind thrombocytopenia are not fully understood but may include immune-mediated platelet destruction, reduced platelet production, hypersplenism, opportunistic infections, malignancies, and the myelosuppressive effects of HIV treatments [16].

Thrombocytopenia in HIV infection can be primary or secondary types; primary thrombocytopenia is more prevalent than secondary thrombocytopenia in HIV infection. Any stage of HIV illness can cause primary HIV-associated thrombocytopenia (PHAT), which in its early stages might mimic immune thrombocytopenic purpura (ITP) because of enhanced platelet breakdown. Immune complexes and antibodies are implicated in this destruction, which could result in faster platelet clearance [17]. Other than HIV infection, conditions like pharmaceutical side effects can also induce secondary thrombocytopenia [17].

Leucopenia is a common hematological disorder in individuals with HIV, caused by HIV itself, autoimmune diseases, cancers, and certain antiretroviral therapies (ART), especially those involving zidovudine [18,19]. Neutropenia, the most prevalent type of leucopenia, impacts 10% to 30% of individuals living with HIV and is associated with reduced cell production resulting from HIV’s influence on the bone marrow. Typically, it is mild, but it can also be severe, increasing the risk of unexpected bacterial infections. Additionally, rare diseases such as aspergillosis or mucormycosis may arise in these patients [20,21]. Studies indicate that lower CD4 counts and advanced HIV disease correlate with increased neutropenia risk. ART enhances the production of important factors like Granulocyte-macrophage colony-stimulating factors(GM-CSF), leading to reduced leucopenia and neutropenia rates in patients receiving treatment [22,23].

Cytopenia represents a significant hematological complication in HIV-infected individuals, irrespective of CD4 count, exhibiting associations with AIDS progression, diminished survival, and serving as an independent predictor of adverse outcomes [2]. While antiretroviral therapy (ART) demonstrably reduces the incidence of cytopenia compared to its absence, the management and consequences of HIV-associated cytopenia diverge significantly between developed and developing nations. Existing evidence reveals mortality rates among HIV-positive individuals in developing countries that are three- to four-fold higher than those observed in industrialized settings. The impact of cytopenia on treatment outcomes and overall survival is particularly pronounced in resource-limited settings, where patients frequently present with advanced disease and treatment-complicating comorbidities [19,24].

Ethiopia, a nation disproportionately affected by the HIV epidemic, reported approximately 603,537 individuals living with HIV, alongside 7,194 new cases and 9,984 HIV-related deaths in 2023, according to EPHI data [25]. The effective management of cytopenia in this context is hampered by a confluence of factors, including limited access to sophisticated monitoring facilities, restricted availability of expert hematological consultation, and persistent financial constraints [24,26,27]. A review of the existing literature indicates a wide range in reported cytopenia prevalence among HIV-infected adults within Ethiopia, spanning from 15% to 60% across various studies. These discrepancies are likely due to study design, sample size, specific populations investigated, and notably, the duration of ART exposure before assessment [16,17,27].

### Rationale and research gap

While multi-institutional studies in resource-constrained settings have established that time-dependent factors significantly influence immuno-hematological profiles during antiretroviral therapy (ART), a substantial knowledge gap remains regarding the prevalence and determinants of cytopenia in Southwest Ethiopia, particularly within the Dawuro Zone. Furthermore, methodological inconsistencies in existing literature have hindered accurate prevalence estimates and the identification of consistent risk factors. This study addresses these limitations by utilizing standardized protocols across multiple public institutions to comprehensively assess cytopenia within a defined timeframe after ART initiation.

### Clinical and Policy Implications

By identifying the specific prevalence and risk factors associated with cytopenia, this research underscores the imperative of integrating routine Complete Blood Count (CBC) monitoring into standard HIV follow-up care. Such integration is critical for enabling timely clinical interventions, reducing cytopenia-related morbidity and mortality, and lowering associated healthcare expenditures. These findings will serve as a vital evidence-based resource for healthcare professionals and policymakers, informing the development of targeted diagnostic strategies and refined ART monitoring protocols to proactively manage hematological abnormalities before they progress to severe complications.

### Scientific contribution and sustainability

Moving beyond traditional opportunistic infection management, this investigation emphasizes the necessity of comprehensive hematological screening in the Ethiopian context. It establishes a crucial scientific baseline for future longitudinal research examining the long-term effects of ART on immuno-hematological health. Ultimately, this study provides a foundational framework for the ongoing monitoring and evaluation of intervention strategies, ensuring the proactive optimization of treatment regimens for people living with HIV.

### Conceptual framework of the study

This conceptual framework was created after a rigorous evaluation of earlier studies. The link between cytopenia and potential influencing factors such as sociodemographic, nutritional, meditational, and clinical factors was shown in the diagram, as indicated in Fig 1.

**Fig 1:**
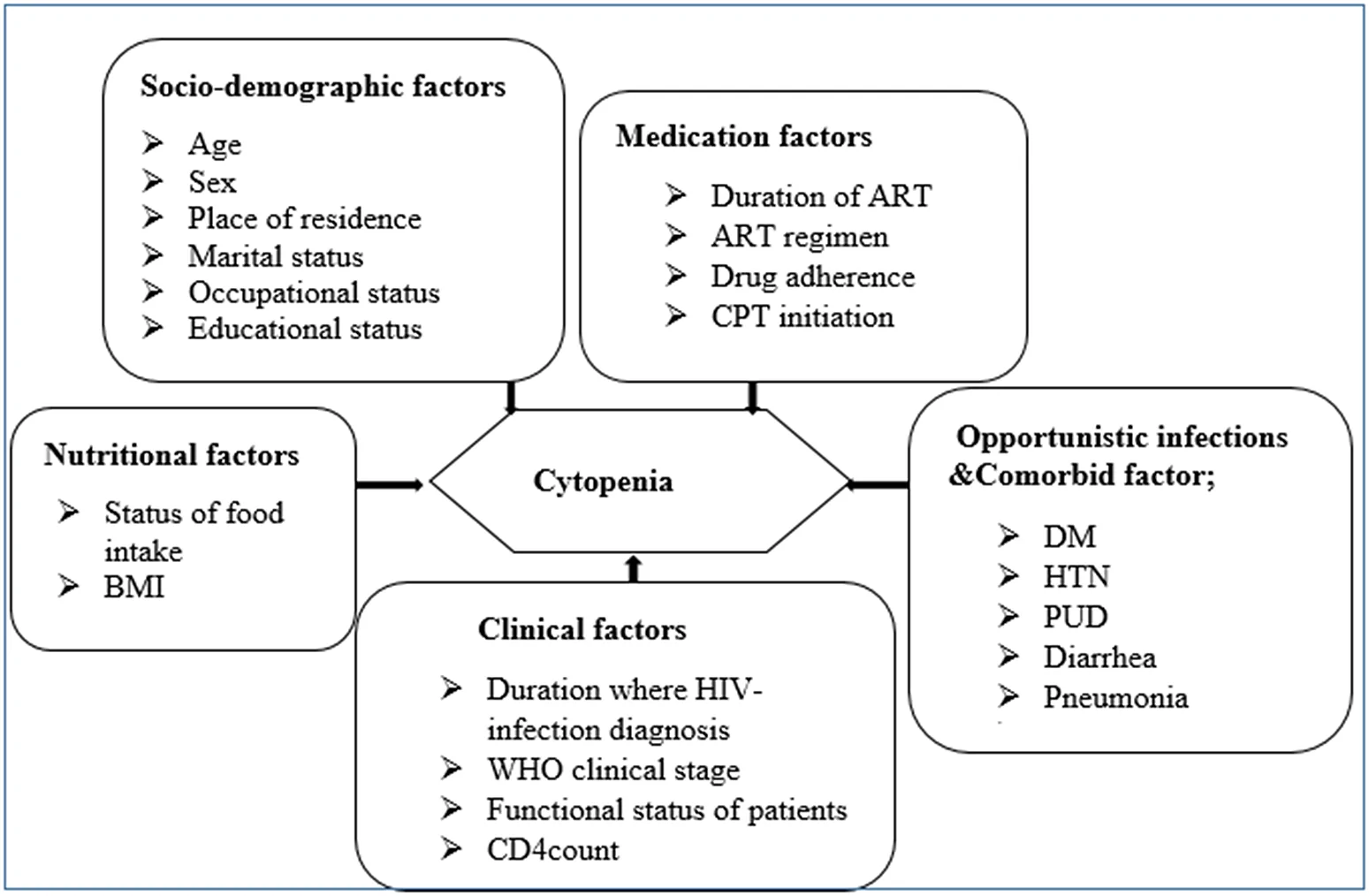
Conceptual Frame work.

## Methods and materials

### Study Area and period

The study was conducted from March 1 to April 30, 2025, in the Dawuro Zone, part of the South West Ethiopia Peoples’ Regional State (SWEPR). The zonal administrative center, Tercha, is located 571 km from Addis Ababa and 285 km from Hawassa. The zone’s health infrastructure includes 186 health posts, 21 health centers, and three public hospitals: Tercha General, Gessa Primary, and Tocha Primary Hospitals. These hospitals serve as the primary ART referral hubs for the zone, providing comprehensive HIV/AIDS care, longitudinal clinical monitoring, and nutritional counseling. According to Federal Ministry of Health indicators and zonal reports, approximately 2,450 active ART patients were registered across these facilities by late 2024 [28]. During the eight-week study period, an estimated 491 patients were scheduled for follow-up at the participating ART clinics.

### Study design

A multi-center, facility-based cross-sectional study was conducted to determine the prevalence, patterns, and associated risk factors of cytopenia among HIV-positive adults on HAART within the public hospitals of the Dawuro Zone.

### Population

**Source Population:** All HIV-positive individuals receiving antiretroviral therapy (ART) at the follow-up clinics of the three public hospitals in the Dawuro Zone.

**Study Population:** All HIV-positive adults who had initiated HAART and attended the targeted ART clinics during the data collection period.

**Sampling frame:** For this study, the frame consists of the chronological sequence of HIV-positive adults scheduled for and presenting at the ART follow-up clinics of the three participating hospitals during the data collection period. The frame’s size was estimated using the 2024 Health Management Information System (HMIS) records, which projected a total of 491 expected clinical encounters for the study duration.

### Eligible criteria

#### Inclusion criteria.

- HIV-positive adults aged >20 years.
- Patients had HAART for greater than 6 months.
- Individuals who were present during the study period and voluntarily gave written informed consent were included.

#### Exclusion criteria.

- Pregnant women.
- Individuals on treatment for any hematological abnormalities during the previous 3 months.
- Patients who received chemotherapeutic agents for six months during the study period.
- Those taking vitamins and iron supplementation for a known hematological problem at the time of sampling.
- Patients who had received a blood transfusion in the last 3 months.
- Patient diagnosed with chronic liver disease and chronic kidney disease.
- Patients who had traumatic injury or surgical interventions resulting in blood loss during the study period or within 3 months before the study period.
- Refused to become part of the study were excluded.

### Sample size determination

The sample size was initially calculated using the single population proportion formula, which is the standard approach for estimating the prevalence of a clinical condition in a large population. Sample size was determined using the single population proportion formula.


$$\begin{array}{c} \frac{{n_{\text{0}}\;=\;Z}^{\text{2}}.p.(\text{1}-p)}{E^{\text{2}}}\;=\frac{.\text{0}.\text{3}\text{9}(\text{1}-\text{0}.\text{3}\text{9})}{{\text{0}.\text{0}\text{5}}^{\text{2}}=365} \end{array}$$

Where:

- ,$n_{\text{0}}$ is the sample size.
- Z is Z- value (for 95% confidence interval Z ≈ 1.960.
- p is the estimated prevalence of cytopenia (39.9%), based on a previous study conducted in a similar Ethiopian clinical setting [19].
- E is the margin of error (the desired level of precision), which is 0.05.

The Finite Population Correction (Reduction Formula) was utilized in this study. Thus, the source population (the number of HIV patients expected for follow-up during the study period, N = 491) was relatively small (less than 10,000). To achieve greater statistical efficiency and accuracy, we applied the Finite Population Correction (FPC) formula to calculate the final sample size, $n=\;\frac{n_{\text{0}}}{\text{1}+\frac{n_{\text{0}}}{N}}$ = $\frac{\text{3}\text{6}\text{5}}{\text{1}+\frac{\text{3}\text{6}\text{5}}{\text{4}\text{9}\text{1}}}$= **210**.

Final Sample Size calculated to account for potential attrition, incomplete laboratory data, or non-response by considering a 10% non-response rate (210*0.1 = 21,210 + 21 = 231), thus, the sample size becomes **231**.[**n = 231**]

### Sampling procedure and techniques

**Sampling procedure** As indicated in Fig 2, a proportional allocation method was employed to ensure that the sample size from each of the three hospitals was representative of its respective patient load. The allocation was based on the Health Management Information System (HMIS) reports from 2024, which identified an expected total of 491 HIV-positive clients scheduled for follow-up during the study period. The total sample size (n = 231) was distributed across the facilities as follows:

**Fig 2:**
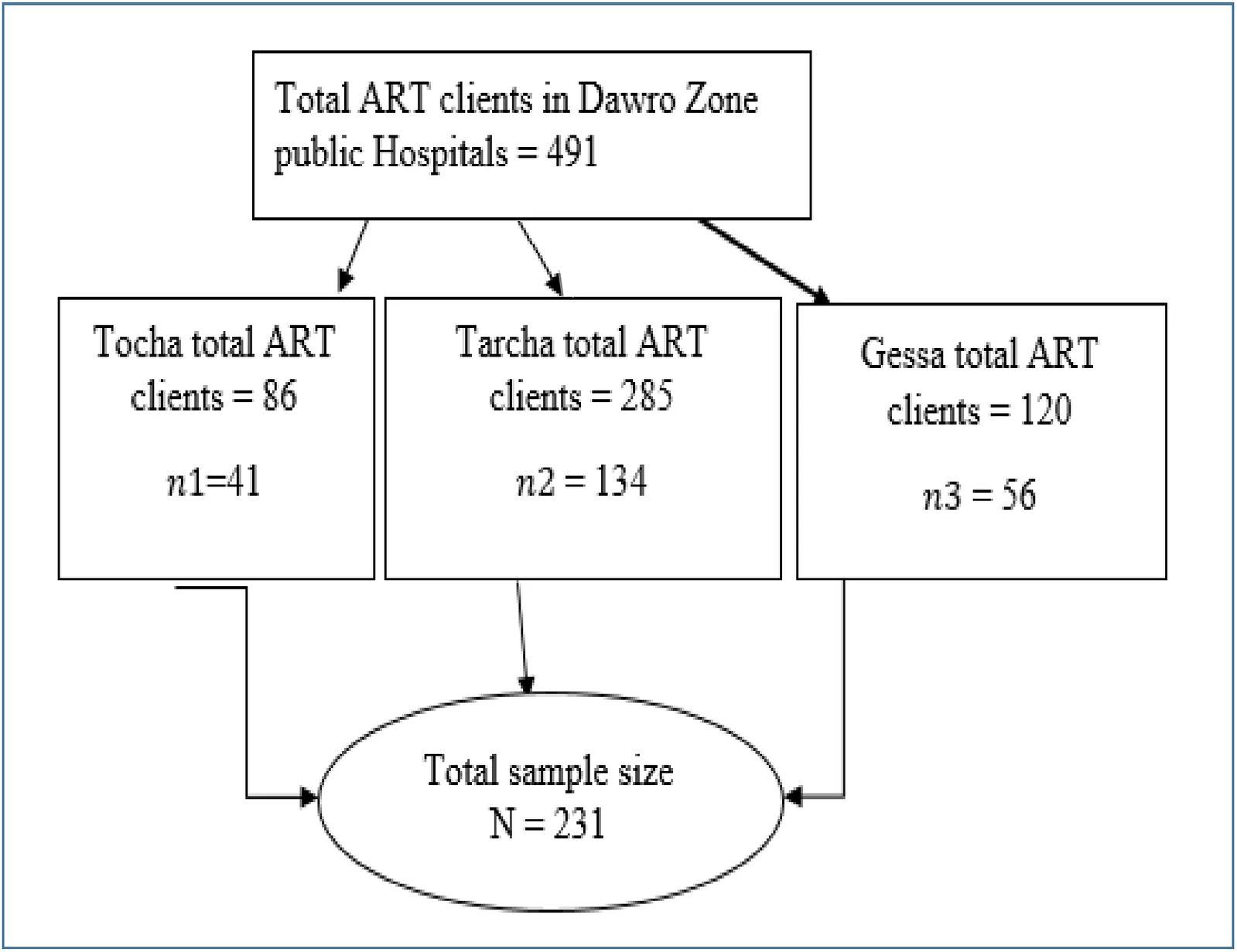
Sampling Techniques.

- Tercha General Hospital: 134 participants (from a follow-up population of 285).
- Tocha Primary Hospital: 41 participants (from a follow-up population of 86).
- Gessa Primary Hospital: 56 participants (from a follow-up population of 120).

**Sampling Technique** A systematic random sampling technique was utilized to recruit study participants. First, a sampling interval (k^th^) was determined by dividing the total expected source population (N = 491) by the final sample size (n = 231), resulting in a k^th^ = N/n, which becomes a value of 2(k^th^ = 2). To initiate the selection process at each facility, a starting point between 1 and k^th^ was determined using a lottery method to select a starting point from the list of clients.

Subsequently, every 2nd patient, starting with the first clients, was recruited throughout the studies based on their arrival at the ART clinic, and were invited to participate in the study. Upon arrival, each selected individual was screened for eligibility based on the pre-defined inclusion and exclusion criteria. This systematic process continued at each site until the proportionally allocated sample size for that specific hospital was achieved.

### Data collection, methods, tools, and procedure

#### Data collection methods.

Data were gathered through a multi-modal approach involving structured interviews, medical chart reviews, physical examinations, and objective laboratory measurements. Following the modern standards of clinical research in 2025, data were captured using internet-based tools created via Kobo Toolbox and collected via Kobo Collect (v2023.2.4) on mobile tablets to minimize entry errors and ensure data integrity.

Trained nurses at the ART follow-up clinics conducted the clinical interviews and anthropometric assessments. Blood pressure and BMI measurements were standardized across all sites. Laboratory technicians performed the venipuncture and subsequent immuno-hematological analysis using automated platforms. In addition, one qualified laboratory technician from each hospital supervised the data collection process, laboratory quality, and also supervised the overall activities during the data collection period.

### Data collection instruments

In order to investigate HIV-associated immuno-hematological complications, we developed a comprehensive and structured questionnaire, drawing upon validated instruments and an extensive review of relevant literature. The data collection tool was meticulously organized into distinct modules to ensure thoroughness and clarity in capturing multifaceted information.

The first module focused on the socio-demographic characteristics of participants, encompassing variables such as age, sex, educational attainment, and geographic residence. The second module addressed clinical and medication histories, including the WHO clinical staging, duration since HIV diagnosis, the presence of opportunistic infections (OIs) or comorbidities, and specific HAART regimens utilized by participants.

The third module collected laboratory parameters, specifically detailing CD4 T-cell counts alongside a comprehensive array of Complete Blood Count (CBC) indices. In the fourth module, anthropometric measurements were recorded, including height, weight, and Body Mass Index (BMI), to assess nutritional status and overall health.

The fifth module examined behavioral and nutritional factors, with a particular focus on substance use (e.g., alcohol and tobacco consumption) and dietary habits. To enhance the academic rigor of our study, we incorporated standardized assessment scales; specifically, we adapted the Morisky Medication Adherence Scale (MMAS) to evaluate adherence to HAART regimens and employed WHO Clinical Staging protocols to accurately categorize disease progression. This structured approach not only facilitates the collection of comprehensive data but also enhances the validity and reliability of our findings, contributing to a nuanced understanding of the immuno-hematological complications associated with HIV.

### Instrument Validity and Reliability

To ensure content validity, the initial questionnaire underwent a rigorous review by a panel of clinical experts and senior researchers specializing in hematology and HIV care. Their feedback was incorporated to ensure the tool accurately captured all relevant clinical and risk factors for cytopenia.

**Pretesting:** A pretest was conducted on 5% of the total sample size at a facility outside the primary study area to evaluate the clarity and cultural appropriateness of the questions.

**Standardization:** All laboratory equipment, including the Beckman Coulter DxH 500 and BD FACSCount™, underwent daily calibration and utilized internal controls according to manufacturer-specific Standard Operating Procedures (SOPs) to ensure high stability and reproducibility of hematological and immunological results.

### Data collection procedure

**Blood sample collection:** Following clients consent, they ordered to give blood sample, just before sample collection, the skin was cleansed, an elastic band (tourniquet) was applied above the area to cause the veins to swell with blood, and four milliliters (4 ml) of venous blood were aseptically extracted into Vacutainer tubes containing plain and ethylene diamine tetra acetic acid (EDTA) for CBC analysis and CD4 T-cell count. During a blood sample collection, blood was drawn from a median cubital vein, usually from the venous arch on the back of the hand or the inside of the elbow. The test took only a few minutes to complete. Before attaching an elastic band or tourniquet to the mid-arm for vein access, technicians used antiseptic hand wipes to clean the patient’s hands before beginning the blood sample collection procedure.

After inserting the needle to draw 4 ml of blood into one or more vials, the elastic band was removed. To prevent further bleeding, the region was wrapped. Blood was then moved to an EDTA tube, and the analysis was performed as soon as possible. The CD4 T-cell count was measured by the BD FACS Count machine (California brand). The hematological parameters were analyzed using an automated hematology analyzer (Beckman Coulter DxH 500 analyzer, a Swiss brand). Ten microliters of EDTA blood were put into the device’s aspirator. Within a minute, the outcome was shown on the LCD screen to be printed. Every laboratory procedure was carried out according to standard operating procedures [29].

All CBC results were interpreted based on the World Health Organization (WHO) definition of hematologic abnormalities [29,30].

- Hb concentration values are low (if Hb < 13 g/dl), normal (between 13-17.5g/dl), and high > 17.5g/dl) for adult males.
- Hb concentration values are low (if Hb < 12 g/dl), normal (between 12-15.5g/dl), and high (>15.5g/dl) for non-pregnant females.
- MCV values low (less than 80fl), normal (between 80 and 100fl), or high (more than that).
- MCHC values low (less than 32 g/dl), normal (between 32 and 36 g/dl), and high (> [31].
- MCH values low (<27pg/cell), normal (between 27 and 32pg/cell), and high (>32pg/cell).
- RBC count low (<4 × 10^6^/μL), normal (4–10 × 10^6^/μL), and high (≥10.1 × 10^6^/ μL).
- WBC count low (<4 × 10^3^/ μL), normal (4–11 × 10^3^/ μL), and high (≥1.1 × 10^3^/ μL).
- Neutrophils value low (less than 40%), normal (between 40% and 60%), or high (>60%).
- The monocyte value is low (less than 2%), normal (2–8%), or high (more than 8%).
- A lymphocyte value is low (<20%), normal (between 20% and 40%), or high (>40%).
- Basophiles value is low, < 0.5%, normal (between 0.5 and 1%), or high (>1%).
- PLT count: Low (<150 × 10^3^/μL), normal (150–450/ μL), and high (>450/ μL) PLT counts.

**The CD4 T cell count** measured by using the BD FACS Count Machine is a critical marker to monitor HIV/AIDS progression and overall immune system health. The results were interpreted based on prior studies [9,19,20].

- CD4 T cell count: Low (< 500 cells/mm^3^), Normal (between 500–1200 cells/mm^3^), and High (>1200 cells/mm^3^), and sever immune deficient (<200 cells/mm^3^).

**Blood pressure [BP]: –** Following 15–30 minutes of rest in a sitting position, from their left arm, while their hands were at the level of their heart, using a mercury sphygmomanometer made by Deko Met Accoson, England, with an appropriate cuff size. In like manner, the typical SBP and DBP of the two readings recorded as hypertension if SBP ≥ 140, as well as DBP ≥ 90 mmHg, or known hypertensives who had been on antihypertensive drugs, were remembered for the class of patients with hypertension unless normotensive (74,75).

### Weighing procedure

Weight was measured using a calibrated beam balance scale following standardized anthropometric protocols. Participants were instructed to remove shoes and heavy outerwear; in instances where specific garments remained, pockets were emptied, and the condition was documented on the data collection form. Subjects stood centrally on the platform with weight evenly distributed until the beam reached equilibrium. All measurements were recorded to the nearest 0.1 or 0.2 kg in accordance with established guidelines [32].

### Height measurement procedure

Height was assessed using a stadiometer with participants standing barefoot and feet together. Proper positioning required the heels, buttocks, calves, and the back of the head to be in contact with the vertical surface. The head was aligned in the Frankfort Horizontal Plane—ensuring the inferior margin of the bony orbit was level with the external auditory meatus—while the participant maintained a forward gaze. The headpiece was lowered to the vertex to compress any hair, and measurements were recorded to the nearest 0.5 cm or millimeter [32].

**Body mass index [BMI]**: – is calculated as [squared height in meters squared divided by deliberate body weight in kilograms]. Participants were assigned to one of the three groups as follows: I. Normal BMI ranges from 18.5–24.59. II. Overweight, BMI = 25 to 29.9 III. BMI (obesity) > 29.9 (157) (76).

### Waist circumference [WC] and Waist-to-hip ratio [WHR]

Measurement of the waist circumference (in centimeters) was taken halfway between the top of the iliac crest and the bottom edge of the last palpable rib. A measuring tape was used to measure the hip circumference (in centimeters) around the broadest part of the buttocks. WHR is computed by dividing the waist circumference by the hip circumference. A WHR above 0.90 for males and above 0.85 for females is a strong indicator of abdominal obesity, and behavioral variables like smoking, alcohol consumption, and khat chewing were also assessed based on the WHO stepwise approach for chronic disease. The recommended cutoff waist circumference was 102 cm (40 inches) for men and 88 cm (35 inches) for women, recommended as a cutoff value for increased waist fat accumulation/health risk (79,80).

### Study variables

#### Dependent Variables.

- **Cytopenia**

#### Independent Variables.

- S**ocio-demographic factors**
  - Age, Sex, Residency, Educational status, Occupation, and Marital status.
- C**linical factors**:
  - WHO clinical stages, Functional status of patients, CD4 counts, and Duration since HIV diagnosis.
- **Medication factors**:
  - ART regimen, Duration of ART use, Cotrimoxazole prophylactic therapy (CPT) initiation, and Drug adherence.
- **Nutritional factors:**
  - Status of food intake and Body mass index (BMI).
- **Comorbidities/Opportunistic Infections:**
  - Diabetic mellitus (DM), Hypertension (HTN**,** Peptic ulcer disease (PUD), Pneumonia and Diarrhea, and TB

### Operational definitions

The definitions of anemia, leucopenia, neutropenia, lymphopenia, and thrombocytopenia are based on the WHO definition of hematologic abnormalities [16,26]).

- Anemia defined as
  - Hgb concentration < 13g/dl for males.
  - Hgb concentration <12 g/dl for females (non-pregnant women).
- Anemia, also categorized as:
  - Mild anemia, if Hgb level is between 11.0–12.9 g/dl for men, and (11.0–11.9 g/dl) for non-pregnant women.
  - Moderate anemia, if Hgb level is between 8.0–10.9 g/dl) for both sexes.
  - Severe anemia, if Hgb is lower than 8.0 g/dl) for both sexes.
- Thrombocytopenia for total platelet count is < 150 × 10^3^/μL
- Thrombocytopenia is categorized as platelet counts:
  - Mild thrombocytopenia, if platelet counts are between 100–150 × 10^3^/μL.
  - Moderate thrombocytopenia, if platelet counts are between 50–100 × 10^3^/μL.
  - Severe thrombocytopenia, if platelet counts are < 50 × 10^3^/μL.
- Leucopenia, if WBC count < 4.0 × 10^3^/μL.
- ART Unit is a unit in hospitals responsible for counseling, investigating, and treating HIV-infected persons with antiretroviral drugs.
- Cytopenia is the presence of one or more of anemia, thrombocytopenia, and/or leucopenia (reduction of one or more blood cells) [19].
- Unicytopenia is defined as the presence of anemia or thrombocytopenia, and/or leucopenia.
- Bi-Cytopenia is defined as the presence of two forms of Cytopenia.
- Pancytopenia is defined as having all three forms of Cytopenia simultaneously.
- HAART is defined as two or more antiretroviral drugs.
- Immunodeficiency is defined as CD4 + T lymphocyte counts < 200 cells/mm3.
- Immunologic classification is categorized into three categories based on CD4 counts using the Centers for Disease Control (CDC) criteria: Stage 1 = CD4 count >500 cells/mm3, Stage 2 = CD4 count between 200 and 499 cells/mm3, and Stage 3 = CD4 count<200 cells/mm3 [31].
- The WHO clinical staging of HIV infection in adults and adolescents was classified into stage 1, stage 2, stage 3, and stage 4 [33].
- Clinical AIDS is defined as a self-reported history of a clinical AIDS-defining condition using the 1993 Centers for Disease Control (CDC) criteria [31].
- Drug adherence is defined as the degree to which a person takes medication as prescribed.
- The status of food intake is defined as the assessment of a person’s food intake throughout/over a defined period.
- Functional status of patients is defined as the extent to which a person can carry out daily activities required for self-sufficiency and well- being.

### Data quality control

Pretesting of the questionnaires was done among 5% (12 participants out of the study area (i.e., Amaya Hospital) before data collection, to ensure logical sequence and skip patterns of the questions, and non-ambiguous or unclear questions were not found. To maintain the consistency of the tool, the questionnaire was first translated from English to Amharic and then back to English by Google Translate. The original and translated questionnaires were compared, and discrepancies were reviewed and resolved accordingly.

In each hospital, two data collectors and one supervisor were trained for one week on the objectives of the study, each variable measurement/questionnaire and its implications, demonstration, and practical session on interviewing, laboratory sample collection, labeling, transportation, storage, and testing procedure as per the standard of testing procedure. They were thoroughly instructed to adhere strictly to the guidelines during the procedure to minimize interpersonal variations. The same data collectors performed the physical assessments to reduce intra-/interpersonal variation.

During the training, detailed precautions as to the quality of reagents, amount, expiry, timing, and duration for test procedures, as well as the quality of laboratory instruments and materials, were emphasized to get quality data. During data collection, the supervisors ensured quality data collection by reviewing all collected data for completeness and taking immediate remedial action accordingly. The same code was used on the patient’s card, on the questionnaire, on the sampled blood, and on the laboratory CBC results and was analyzed according to its respective code. Every patient was registered only once in the study. To ensure this, the charts of the patients were included in the study and marked with a sign.

### Data analysis processing

After the data collection period was completed, data were entered, cleaned, and coded using Epi 3.1, then exported to SPSS software version 26 for Windows for analysis, and checked and controlled for any missing values and cleaned from unnecessary data or categories. Variables were coded, recoded, re-categorized, and transformed just before the data analysis phase.

After controlling the data using various data management methods, data analysis was performed using SPSS version 26. Before inferential analyses, rigorous data management procedures were implemented to ensure data quality and accuracy. Descriptive statistics, including frequency distributions, were generated to summarize the characteristics of the study population and to present univariate analyses of both numerical and categorical variables. Frequency tables, graphs, and percentages were used to provide a clear and concise presentation of the data, including the prevalence of Cytopenia, and the distribution of potential predictor variables.

In addition, to determine the independent associations between these and other factors with cytopenia and to control for potential confounders, we constructed a series of multivariate binary logistic regression models (87). Based on results from the bivariate analyses and supporting evidence from prior literature, we included pre-identified key covariates known to influence the risk of Cytopenia. To ensure this model’s validity, assumptions of logistic regression, including the absence of multicollinearity and the presence of adequate model fit, were tested. Multicollinearity was assessed using Variance Inflation Factors (VIFs), with VIF values below a predetermined threshold of 5 indicating minimal concern. To further confirm the robustness of our models, Model fitness was evaluated using the Hosmer–Lem show goodness-of-fit test, and statistical significance was defined as a p < 0.05.

Beginning with a full model including all potential confounders, we sequentially removed variables, starting with the least statistically significant, until all remaining variables exhibited statistically significant associations with cytopenia (p < 0.05), using a 95% confidence interval for hypothesis testing. Adjusted odds ratios (AORs) with corresponding 95% confidence intervals (CIs) were calculated and reported to represent the strength and direction of associations. Following this analysis, findings were discussed, summarized, and recommendations were proposed.

### Ethical considerations

Ethical clearance and approval were granted by the Institutional Review Board (IRB) of the Institute of Health, Jimma University (Protocol No: JUIH/IRB/215/2025). Formal letters of permission were subsequently obtained from the administrative offices of each participating hospital. The study was conducted in accordance with the ethical principles of the Declaration of Helsinki.

Prior to enrollment, the purpose, procedures, and potential benefits of the study were clearly explained to each participant in their local language. Consequently, written informed consent was obtained from all study participants. To ensure strict confidentiality and anonymity, all personal identifiers were removed from the data collection tools and replaced with unique alphanumeric codes. Data were stored securely, and access was restricted solely to the research team.

### Plan for dissemination of results

Study results were submitted to Jimma University and communicated to the Dawuro Zone Health Department and local hospital management to facilitate evidence-based clinical interventions. The findings will be disseminated further through publication in international peer-reviewed journals to contribute to the broader scientific and public health discourse.

## Result

### Sociodemographic characteristics of study participants

The study included 231 participants with response rates of 99.6% (230). The respondent’s mean age was 36 ± 27 years. Age distribution showed that 56 (24.3%) were between 20 and 29 years, and the majority of the participants were female 128 (55.7%), 135(58.7%) lived in urban areas, 130 (56.5%) were married, and 93 (40.4%) were in secondary school and above; with respect to occupational, 51(22.2%) were government employees as shown on Table 1.

**Table 1:** Socio-demographic Characteristics of HIV-Positive Adults on HAART Attending ART Follow-Up Clinics at Dawuro Zone Public Hospitals, Southwest, Ethiopia, 2025 [N = 231].

Variables	Category	Frequency (n = 230)	Percentage (%)
Age (in years)	20-29	56	24.3
	30-39	78	33.9
	40-49	61	26.5
	≥50	35	15.2
Sex	Male	102	44.3
	Female	128	55.7
Residence	Urban	135	58.7
	Rural	95	41.3
Marital status	Married	130	56.5
	Single	81	35.2
	Others^*^	19	8.3
Educational status	Uneducated	49	21.3
	Primary	88	38.3
	Secondary & above	93	40.4
Occupational status	Government employs	51	22.2
	Merchant	48	20.9
	Farmer	44	19.1
	House wife	27	11.7
	Others**	60	26.7

Others* = widowed and divorced, Others** = daily labors, private employer, students

### Clinical, nutritional, medication, and hematological characteristics of the study participants

As summarized in Table 2 below, the clinical and therapeutic profiles of the participants (N = 231) are summarized in Table 2. Regarding functional status, (n = 178,77.4%) participants were categorized as “working,” and the majority (n = 134, 58.3%) were in WHO Clinical Stage I. Opportunistic infections (OIs) were identified in (n = 23,10.0%) participants, with the most frequent being diarrhea (11, 47.8%), followed by pneumonia (n = 6, 26.1%) and tuberculosis (n = 6, 26.1%). Additionally, chronic comorbidities were reported in (n = 29, 12.6%) participants, primarily consisting of hypertension (n = 12, 41.4%) and diabetes mellitus (n = 7, 24.1%).

**Table 2:** Clinical, Nutritional, Treatment, and Hematological Characteristics of HIV-Positive Adults on HAART at ART follow-up clinics of Dawuro Zone Public Hospitals, Southwest, Ethiopia, 2025 (N = 231).

Variables	Category	Frequency	Percentage (%)	Mean (SD)
**Functional status**	Working	178	77.4	
	Ambulatory	39	17	
	Bed ridden	13	5.7	
**WHO Clinical stage of HIV**	Stage1	134	58.3	
	Stage2	65	28.3	
	Stage3	22	9.6	
	Stage4	9	3.9	
**Opportunity infection**	Yes	23	10.0	
	No	207	90.0	
**Types of Opportunity infection (n = 23)**	Diarrhea	11	47.8	
	Pneumonia	6	26.1	
	TB	6	26.1	
**Chronic diseases**	Yes	29	12.6	
	No	201	87.4	
**Type of chronic diseases (n = 29)**	DM	7	24.1	
	HTN	12	41.4	
	Others	10	34.5	
**Current ART regimens**	DTG-based regimen	205	89.1	
	ATV-based regimen	9	3.9	
	LPV/r-based regimen	16	7.0	
**Drug adherence**	Good	132	57.4	
	Medium	27	11.7	
	Poor	71	30.9	
**CPT initiation**	Present	173	75.2	
	Absent	57	24.8	
**Duration of ART regimen (in months)**	<12 months	20	8.7	
	12-59 months	136	59.1	
	>=60 months	74	32.2	
**BMI**	Underweight	59	25.7	
	Normal	129	56.1	
	Overweight	26	11.3	
	Obese	16	7	
**CD4 count cells/mm** ^**3**^	<200 cell/mm^3^	60	26.1	
	200-499 cell/mm^3^	88	38.3	
	>500 cell/mm^3^	82	35.7	
**Status of food intake**	Adequate	178	77.4	
	Decreased	35	15.2	
	Very decreased	17	7.4	

WHO = World Health Organization, ART = antiretroviral therapy, SD = standard deviation, BMI = body mass index, CPT = Cotrimoxazole prophylactic therapy, DM = diabetes mellitus, HTN = hypertension, others = peptic ulcer disease and bronchial asthma, 1j = TDF-3TC-DTG, 2H = TDF-3TC-ATV, 2G = TDF-3TC-LPV/r, TDF = Tenofovir, 3TC = Lamivudine, DTG = Dolutegravir, LPV = Lopinavir, ATV = Atazanavir, r = Ritonavir

Regarding medication and adherence, the vast majority of participants (n = 205, 89.1%) were on a Dolutegravir (DTG)-based regimen, and (n = 173, 75.2%) had initiated Co-trimoxazole Prophylaxis Therapy (CPT). Over half of the study population (n = 132, 57.4%) demonstrated good drug adherence. In terms of treatment duration, (n = 136,59.1%) participants had been on ART for 12–59 months. Nonetheless, nutritional assessment revealed that 25.7% of the participants were underweight (BMI < 18.5 kg/m^2^). Immunological data indicated that only 35.7% of the cohort achieved a CD4 count >500 cells/mm^3^, while 26.1% remained severely immunocompromised with a CD4 count <200 cells/mm^3^, as shown in Table 2.

### Hematological Indices of Study Participants

The hematological and immunological profiles of HIV-positive adults on HAART are summarized in Table 3. Participants exhibited a wide range of CD4 + T-cell counts, from 19 to 1109 cells/mm^3^. The mean (± standard deviation, SD) values for key hematological parameters were: the subjects studied had a baseline CD4 + T cells count ranging from 19–1109 cell/mm^3^ and mean values as follows: CD4 + T cells was 424.14 ± 271.7), hemoglobin concentration was 13.6 g/dL ± 2.61), WBC count was 6.3 × 10^3^/μL ± 2.2 × 10^3^), and platelet count was 250.2 × 10^3^/μL ± 84.9 × 10^3^)

**Table 3:** Hematological Indices of HIV-Positive Adults on HAART in ART Clinics in Dawuro Zone Public Hospitals, Southwest, Ethiopia 2025 [N = 231].

Parameter	Mean ± SD	** reference Range
**Hgb (g/dL)**	13.6 ± 2.61	12–15.5 for females 13–17.5 for males
**WBC Count (×10³/μL)**	6.3 ± 2.2	4.0–11.0 for both sexes
**Platelet Count (×10³/μL)**	250.2 ± 84.9	150–450 for both sexes
**Neutrophil Count(×10³/μL)**	3.4 ± 2.04	2.5-8 for both sexes
**CD4 count cells/ mm** ^**3**^	424.14 ± 271.7	500-1200 for both sexes

** Reference range taken from [29,30].

### Prevalence and pattern of cytopenia among HIV-positive adults

The overall prevalence of cytopenia among HIV-positive adults on HAART in the Dawuro Zone was found to be 27.8% (n = 64; 95% CI: 22.1–34.3%). Further stratification revealed that anemia was the most frequent cytopenic abnormality, occurring in (n = 41,17.8%) participants, followed by leukopenia in 31 (13.5%) and thrombocytopenia (n = 29,12.6%) of the population as per Fig 3.

**Fig 3:**
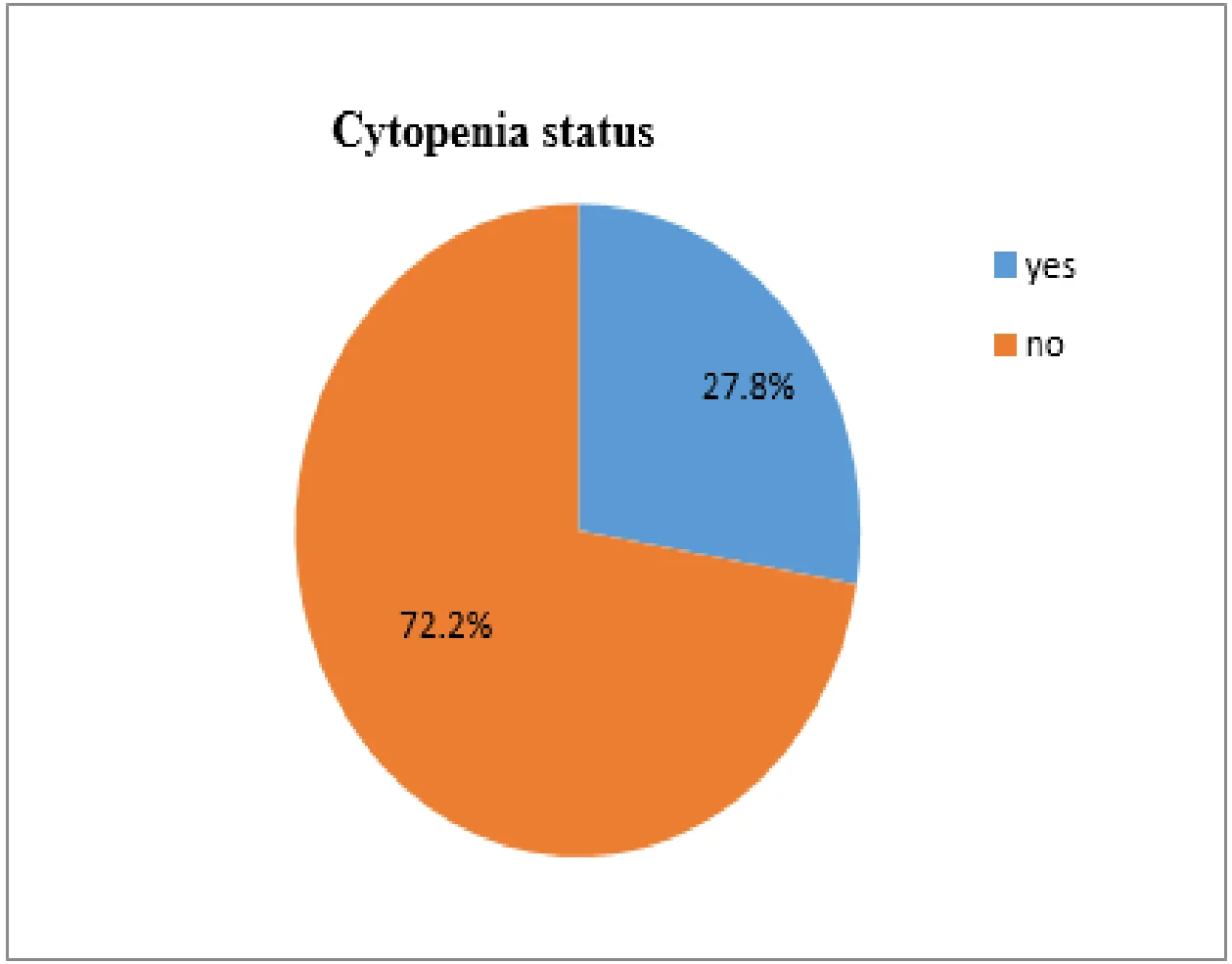
Cytopenia Figures.

An examination of the cytopenic patterns indicated that single-lineage cytopenia (unicytopenia) was the most common manifestation, identified in (n = 34,14.8%) participants. Bi-cytopenia was observed in (n = 23,10.0%) individuals, while pancytopenia was less frequent, affecting (n = 7,3.0%) participants. Within the unicytopenic group, isolated anemia constituted the most prevalent form (n = 15; 6.5%). In the bi-cytopenic cohort, the combination of anemia and leukopenia emerged as the most frequent pattern, accounting for (n = 13,5.7%) of the total study population. The severity of these cytopenic conditions varied by lineage. Among the 41 participants with anemia, the majority (41.5%) presented with moderate anemia. In contrast, a substantial majority of participants with thrombocytopenia (79.3%) exhibited a mild form of the condition. These detailed classifications of cytopenic patterns and their respective severities are further in Table 4.

**Table 4:** Pattern Of Cytopenia Among HIV-Positive Adults on HAART In ART Clinics of Dawuro Zone Public Hospitals, Southwest, Ethiopia, 2025 [N = 231].

Pattern of cytopenia	Frequency(n)	Percentage (%)
Isolated anemia	15	6.5
Isolated leucopenia	7	3
Isolated thrombocytopenia	12	5.2
Bi-cytopenia		
Anemia and leucopenia	13	5.7
Anemia and thrombocytopenia	6	2.6
Thrombocytopenia and leucopenia	4	1.7
Pancytopenia	7	3.0
Anemia severity (n = 41)		
Mild	15	36.6
Moderate	17	41.5
Severe	9	22.0
Thrombocytopenia severity (n = 29)		
Mild	23	79.3
Moderate	6	20.7
Severe	0	0

### Factors associated with cytopenia among HIV-infected adults

#### Bivariate analysis of factors affecting cytopenia.

Binary logistic regression was done for each factor associated with cytopenia among HIV-infected adults, as listed below in Table 5. Bivariate analysis was used to examine the relationship between socio-demographic, clinical, medication, and nutritional variables with cytopenia. Among the variables that were candidate variables for the multivariable logistic regression model with a p-value less than 0.25 were age, sex, BMI, functional status, duration of ART regimen, WHO clinical stage HIV infection, opportunistic infection, chronic diseases, drug adherence, CPT initiation, and CD4 count.

**Table 5:** Bivariate and multivariate analyses of factors to determine independent factors associated with cytopenia among HIV-positive adults on HAART in ART clinics of Dawuro Zone Public Hospitals, Southwest, Ethiopia, 2025 (N = 231).

Variables	Cytopenia		Bivariable	P-value	Multivariable	P-value
	Yes, n (%)	No, n (%)	COR (95% CI)		AOR (95% CI)	
**Age**						
20-29	11(19.6)	45(80.4)	1		1	
30-39	24(30.8)	54(69.2)	1.818(.80-4.11)	.151	3.35(.99-11.3)	0.051
40-49	14(23.0)	47(77.0)	1.219(.50-2.97)	.663	1.9(.508-6.9)	0.35
≥50	15(42.9)	20(57)	3.068(1.2-7.9)	.019	5.1(1.3–2.1)	.023*
**Sex**						
Male	37(36.3)	65(63.72)	2.13(1.2-3.8)	0.01	2.82(1.23-6.46)	0.014*
Female	27(21.09)	101(78.9)	1	1		
Residence						
Urban	34(25.2)	101(74.8)	1	1		
Rural	30(31.6)	65(68.4)	1.37(.76-2.45)	.288		
**Marital status**						
Married	36(27.6)	94(72.3)	.83(.29-2.35)	.725		
Single	22(27.2)	59(72.8)	.80(.27- 2.38)	.700		
Others *	6(31.6)	13(86.4)	1	1		
**Educational status**						
Uneducated	15(30.6)	34(69.4)	1.13(.53-2.42)	.740		
Primary	23(26.1)	65(73.9)	.91(.47-1.75)	.783		
Secondary & above	26(28)	67(72)	1			
**Occupational status**						
Government employs	14(27.4)	37(72.6)	1.4(.58-3.3)	.54		
Merchant	18(37.5)	30(62.5)	2.2(.91-5.09)	.73		
Farmers	13(29.5)	31(70.5)	1.52(.62-3.7)	.4		
House wife	6(22.2)	21(77.8)	1.03(.35-3.1)	.95		
Others**	13(21.7)	47 (78.7)	1			
**Functional status**						
Working	42(23.6)	136(76.4)	1			
Ambulatory	14(35.9)	25(64.1)	1.813(.87-3.8)	.115	2.04(.723-5.74)	.178
Bed ridden	8(60.5)	5(38.5)	5.181(1.61-16.7)	.006	5.95(1.31-27.15)	.021*
**WHO stage**						
Stage1	32(23.9)	102(76.1)	1			
Stage2	18(27.7)	47(72.3)	1.221(.623-2.39)	.561	2.04(.723-5.74)	.680
Stage3	1150)	11(50)	3.187(1.26-8.04)	.014	1.06(.25-4.56)	.94
Stage4	6(60)	3(30)	1.594(0.377-6.74)	.526	1.17(.132-10.39)	.887
**Opportunistic infection**						
Yes	14(60.9)	9(39.1)	4.88(1.99-11.9)	.001	4.57(1.14-18.37)	.032*
No	50(24.1)	157(75.8)	1			
**Chronic diseases**						
Yes	15(51.70	14(48.3)	3.32(1.49-7.37)	.003	1.83(.56-5.89)	.32
No	49(24.4)	152(75.6)	1	1		
**Types of chronic diseases**						
DM	5(71.4)	2(28.6)	3.75(.47-29.75)	.251		
HTN	5(45.5)	6(55.5)	1.5(.27-8.18)	.640		
Others	5(45.5)	6(55.5)	1	1		
**ART regimen**						
DTG-based regimen	49(23.7)	156(75.4)	1		1	
ATV-based regimen	4(44.4)	5(55.6)	2.54(.65-9.85)	.176	.79(.15-6.44)	.98
LPV/r-based regimen	11(68.8)	5(31.3)	7.0(2.32-21.14)	.001	6.06(1.39-26.56)	.017*
**Drug adherence**						
Good	21(15.9)	111(84.0)	1			
Medium	5(18.5)	22(82.5)	1.2(.40-3.52)	.739	.74(.17-3.21)	.69
Poor	38(53.5)	33(46.5)	6.08(3.14-11.7)	.000	6.97(2.92-16.64)	.000*
**CPT**						
Present	44(25.4)	129(74.6)	1			
Absent	20(35.09)	37(64.)	1.58(.83-3.01)	.160	.88(.31-2.51)	.81
**Duration of ART**						
<12	2(10)	18(90)	1			
12-59	35(25.7)	101(74.3)	3.11(.68-14.12)	.140	1.44(.25-8.44)	.69
>=60	27(36.5)	47(63.5)	5.17(1.11-24.0)	.036	1.40(.23-8.70)	.72
**BMI**						
Underweight	22(37.3)	37(62.7)	1			
Normal	29(22.5)	100(77.5)	.488(.249-.953)	.036	.49(.81-1.29)	.15
Overweight	8(30.8)	18(69.2)	.747(.279-2.003)	.563	1.49(.39-5.77)	.56
Obese	5(30.8)	11(68.8)	.764(.235-2.492)	.656	1.20(.23-6.33)	.826
**CD4count cells/mm^3^**						
<200	26(43.3)	34(56.7)	6.203(2.62-14.7)	.000	3.45(1.05-11.39)	.042*
200-499	29(32.95)	59(67.05)	3.987(1.75-9.08)	.001	3.36(1.18-9.57)	.023*
>500	9(11.0)	73(89.02)	1			
**Status of food intake**						
Adequate	51(28.7)	127(71.4)	1			
Decreased	8(28.9)	27(77.1)	.73(.31-1.73)	.485		
Very decreased	5(29.4)	12(70.6)	1.0(.34-3.09)	.947		

* Shawn statistical significance AOR = adjusted odd ratio, COR = crude odd ratio, CI = confidence interval, 1 = reference group.

#### Multivariate analysis of factors affecting cytopenia.

The model’s fitness was assessed using the Hosmer and Lemeshow test, indicating evidence for model fitness. Old age, male gender, functional status of patients, opportunistic infection, LPV/r-based ART regimen, poor drug adherence, and CD4 count category 200–499 cells/mm3 and CD4 count <200 cells/mm3 were significantly associated with cytopenia (p ≤ 0.05) (as shown in Table 5). Those who were older than 50 years had 5.13 times the risk of cytopenia as compared to those who were aged 20–29 years [AOR = 5.13: CI; 1.25–21.07, P = 0.023]. The risk of cytopenia was 2.82 times higher among males’ gender than females [AOR = 2.82, CI: 1.23–6.46, P = .014]. Those who were bedridden patients were 5.95 times more likely to have cytopenia than those who were working [AOR = 5.95, CI: 1.31–27.15, P = .021].

Those who had opportunistic infection were at 4.57 times the risk of cytopenia as compared to those who had non-opportunistic infection [AOR = 4.57, CI: 1.14–18.37, P = 0.032]. The risk of cytopenia was 6.06 times higher in patients who were on an LPV/r-based regimen as compared to those who were on a DGV-based regimen [AOR = 6.06, CI: 1.39–26.56, P = 0.017], and those who had poor drug adherence were 6.97 times as likely to have cytopenia as those who had good drug adherence [AOR = 6.97, CI: 2.92–16.64, P = 0.000]. Moreover, those who had less than 200 cells/mm3 of CD4 count were 3.45 times more likely to be cytopenia than those who had greater than 500cells/mm3 of CD4 counts [AOR = 3.45, CI: 1.046–11.39, P = 0.042] and those whose CD4 count category 200–499 cells/mm3 were 3.36 time risk of cytopenia than those who had greater than 500 cells/mm3 [AOR = 3.36, CI: 1.18–9.57, P = 0.023] seen in Table 5.

## Discussion

### Prevalence of Cytopenia

This study assessed the prevalence and associated factors of cytopenia among HIV-positive adults receiving Highly Active Antiretroviral Therapy (HAART) in public hospitals within the Dawuro Zone, Ethiopia. The overall prevalence of cytopenia was 27.8% (95% CI: 22.1–34.3%). This finding is notably lower than the prevalence rates reported in previous studies conducted in the North Shewa Zone, Ethiopia (39.9%), Nigeria (84%), and Uganda (65%) [19,24,26]. These differences could be due to differences in current ART management protocol, use of cut-points to define cytopenia, methodology, study population, healthcare access and utilization, socioeconomic status, dietary habits of study participants, and healthcare service quality.

The potential mechanisms by which HIV may influence the bone marrow include the possibility of direct infection of stromal cells, which could disrupt the microenvironment necessary for hematopoiesis. An imbalance in cytokines, such as interleukin-1 (IL-1), interferon-gamma (IFN-γ), and tumor necrosis factor-alpha (TNF-α), has been observed during HIV infection and may be associated with impaired proliferation and differentiation of blood cell precursors. Additionally, immune dysregulation resulting from HIV infection might contribute to alterations in bone marrow function. Furthermore, certain medications used to treat HIV or related conditions have been reported to potentially affect bone marrow activity [34].

### Pattern Of cytopenia

In this study, we found that the most common type of cytopenia was unicytopenia (n = 43; 14.8%), followed by bi-cytopenia (n = 23; 10%), and pancytopenia (n = 7; 3%). Our findings show that bi-cytopenia is the second most common type of cytopenia, which is in line with a previous study done in Ethiopia (11.6%) [19]; however, this finding is lower than studies done in Nigeria (27%) and Uganda (21.4%). The possible cause could be differences in study population characteristics, stage of HIV infection, and access to ART drugs.

Our finding also shows that pancytopenia, another common type of cytopenia, agrees with a previous study done in Nigeria (3%), but is higher than the studies conducted in Ethiopia (1%) and Uganda (2%), and lower than the study done in Beijing, China (5.7%) [19,24,26,34]. This variation could be attributed to differences in the inclusion criteria of study participants, sample size, stage of disease progression, nutritional status of patients, prevalence of co-infections, and health care access among the study populations.

Among unicytopenia, anemia was the most common cytopenia, and anemia and thrombocytopenia were the most common bi-cytopenia. Of the anemic study subjects (n = 41,22%) had severe anemia, 41.5% had moderate anemia, and 36.6% had mild anemia. The predominance of moderate types of anemia in the current study, in line with studies done in the Gedeo Zone of southern Ethiopia [35] and Beijing, China [36], is contrary to a longitudinal cohort study done in America, which showed that the predominant types of anemia are mild anemia [37]. The observed difference is due to better healthcare coverage, earlier detection via routine screening, and improved nutritional support. According to the levels of severity, mild thrombocytopenia was the most common, which is in line with Gondar, Ethiopia [3], and North Ethiopia [19]. The observed similarity is due to common sociodemographic characteristics, nutritional support, and the cut point to define cytopenia.

### Factors Associated with Risk Factors of Cytopenia

Our study findings showed that being old (age ≥ 50) is more likely to cause cytopenia than being young [AOR = 5.13: CI; 1.25–21.07, P = 0.023]. This finding is in harmony with a cross-sectional study done by Gebreweld *et al* [16]. They found that older HIV-positive adults had 0.3 times more prevalence than young adults (p-value = 0.004). This finding was also supported by another study done in Nigeria, which found HIV-positive adult patients who were old showed a significant association with the progression of cytopenia (p-value = 0.01) as compared with those young adults [26]. The observed phenomenon may be related to the natural reduction in immune function and bone marrow regenerative capacity that occurs with aging. This decline could be further influenced by chronic viral infections, which might complicate the process of producing new blood cells compared to younger adults [38].

This study showed males are 3 times more likely to have cytopenia compared to females [AOR = 2.82, CI: 1.23–6.46, P = .014]. This finding is similar to a previous study done in Ethiopia, which found that HIV-positive adult patients [19]. However, our result is contrary to another finding done in Beijing, China [34] and Uganda [24], which reported that cytopenia is more likely to occur among females than in males. One possible explanation for the differences in cytopenia between sexes could involve the cut-point used to define it and hormonal variations. Estrogen, primarily found in females, has immune-modulating and anti-inflammatory properties that may protect bone marrow progenitor cells, reducing the risk of cytopenia. In contrast, testosterone, more prevalent in males, promotes erythropoiesis but may increase susceptibility to bone marrow suppression under stress or chronic illnesses like HIV. These interactions require further investigation [39].

On the other hand, our findings confirmed that the bedridden functional status of the patient is significantly associated with cytopenia than that of working participants [AOR = 5.95, CI: 1.31–27.15, P = .021]. This finding is not addressed by other studies. The possible explanation is that the patients with HIV who are bedridden usually reflect an advanced stage of the disease’s course, marked by severe immunosuppression, systemic inflammation, opportunistic infections, and inadequate nutrition. The development of HIV-induced cytopenia, such as anemia, leukopenia, and thrombocytopenia, is significantly influenced by these clinical characteristics [40].

Moreover, this study found that the presence of opportunistic infection was more significantly associated with cytopenia [AOR = 4.57, CI: 1.14–18.37, P = 0.032]. This finding is concurrent with studies conducted in Beijing, China, as they reveal HIV-positive adult patients who have opportunistic infection showed a 5 times higher chance of developing cytopenia than those without at (p-value = 0.01) [34]. Our finding is in sharp contrast with those of others found, who have found that having an opportunistic infection had no significant association with cytopenia [19,24]. This could be because of opportunistic infections that can directly or indirectly invade or suppress bone marrow cells and impair the bone marrow’s capacity to generate blood cells, which finally, it leads to different hematologic complications [40].

Our study also found that cytopenia was six times more likely to occur among those on the LPV/r-based regimen as compared to those patients who were on a DGV-based regimen [AOR = 6.06, CI: 1.39–26.56, P = 0.017]. This finding is supported by another study done in Nanning, China, which found HIV-positive adult patients who were on an LPV/r-based regimen showed a significant association with cytopenia (p-value = 0.01) [41]. The significant association observed between LPV/r-based regimens and cytopenia is consistent with the literature, suggesting a distinct myelosuppressive footprint for certain Protease Inhibitors. This effect may be exacerbated by the overproduction of reactive oxygen species (ROS), which induces oxidative damage and mitochondrial dysfunction within the bone marrow microenvironment. Such oxidative stress is known to impair the survival and proliferative capacity of hematopoietic stem and progenitor cells (HSPCs), thereby reducing effective hematopoiesis [42].

Furthermore, beyond these molecular mechanisms, the clinical association observed here may be influenced by the gastrointestinal adverse effect profile of LPV/r, which is frequently linked to the malabsorption of essential micronutrients required for blood cell synthesis. These findings align with current therapeutic trends favoring Integrase Strand Transfer Inhibitors (INSTIs) like Dolutegravir, which demonstrate superior hematological safety profiles compared to older PI-based regimens, likely due to their minimal interference with mitochondrial and ER homeostasis [43].

Suboptimal ART adherence likely drives persistent viral replication and immunodeficiency, potentially disrupting the bone marrow microenvironment and increasing susceptibility to opportunistic infections that further impair hematopoiesis. This chronic viral presence may stimulate the overproduction of pro-inflammatory cytokines (e.g., TNF-α, IFN-γ, and IL-6), which are suggested to induce cell cycle arrest in hematopoietic progenitor cells via the NF-κB and JAK/STAT signaling pathways. By upregulating cell cycle inhibitors such as p21 and p27 while suppressing essential cyclins, these inflammatory mediators likely hinder the proliferation and differentiation of blood cell precursors, ultimately providing a probable biological mechanism for the multi-lineage cytopenia observed in patients with poor treatment adherence [44,45].

Our study also revealed that study participants with a CD4 T cell count less than 200 cells/mm^3^ were more likely to have cytopenia than those with a CD4 count greater than 500 cells/mm3 [AOR = 3.45, CI: 1.046–11.39, P = 0.042]. This finding is consistent with a cross-sectional study done by Gebreweld et al [19], as they found that HIV-positive adults whose CD4 count was below 200 cells/mm³ were 6 times more likely to have cytopenia than those whose CD4 count was greater than 500 cells/mm³ (p-value = 0.001). This finding was also supported by another study done in Uganda and Beijing, as they found that HIV-positive adult patients whose CD4 count was below 200 cells/mm³ showed a significant association with the progression of cytopenia at (p-value = 0.001) and (p-value = 0.013), respectively, as compared with those whose CD4 count was greater than 500 cells/mm³ [24,34]. The possible explanation is that when the CD4 + T-cell counts drop, the immune system’s effectiveness decreases, increasing the risk of various opportunistic infections that can directly or indirectly invade or suppress bone marrow cells and impair the bone marrow’s capacity to generate blood cells, which leads to different hematologic and related problems [40].

In addition, in people with CD4 T cell counts under 200 cells/mm³, the immune system stays active for a long time, mainly due to ongoing HIV replication and co-infections like CMV, EBV, and TB. This sustained immune activation affects key immune cells, particularly T cells and monocytes/macrophages. As a result, there is an overproduction of pro-inflammatory cytokines that negatively impact the bone marrow, inducing progenitor cell death, altering the bone marrow microenvironment, and impairing the synthesis and differentiation of blood cells. Additionally, peripheral cytopenia can occur as these cytokines stimulate macrophages and other immune cells to phagocytose circulating blood cells [46,47]

### Limitation of the study

In this study, the primary limitation is its cross-sectional design, which does not identify causal relationships. To generalize the findings of this study, a longitudinal investigation is advised. We were unable to evaluate the direct relationship between viral load and cytopenia since the facility lacked viral load testing kits. Despite the limitations, the study has assessed the prevalence of cytopenia and identified significant risk variables for cytopenia in HIV-positive adult patients on HAART.

## Conclusion and recommendation

### Conclusion

In conclusion, among HIV-positive individuals receiving HAART, the prevalence of cytopenia was 27.8%, indicating that while cytopenia falls within the midrange of reported values from other previous studies, and still a concern. Even if its burden in this cohort is relatively moderate. Unicytopenia was the most common kind of cytopenia, followed by bi-cytopenia. Older age group greater than 50 years, male gender, Opportunistic infections, LPV/r-based ART regimen, poor drug adherence, and CD4 count less than 200 cells/mm³ were factors associated with cytopenia.

### Recommendation

Based on the findings of the study, the following recommendations are given.

### For Dawro Zone public Hospitals and health workers

- We recommend that hospital staff identify and approach cytopenia based on the mentioned modifiable associated factors to improve cytopenia.
- Additionally, medical professionals who treat HIV-positive patients should address the negative effects of LPV/r-based ART regimens and manage them accordingly.
- We also recommend that health professionals provide health education on preventing infectious diseases, medication adherence, and early intervention of infections.
- A more frequent screening of cytopenia and early clinical interventions.

### For other researchers

- It is better to conduct a further study with a longitudinal study to investigate the cause-effect relationship of associated factors and cytopenia.
- We advise future researchers to incorporate viral load measurement and enable them to assess the effect of viral burden on the onset and progression of cytopenia.

### For policymakers

- Cytopenia is quite prevalent in this cohort; thus, screening for cytopenia and addressing the problem to improve treatment outcomes for the patient.
- Ensure healthcare provider training on the identification and management of ART-related hematological complications to improve patient care at all ART clinics.

## Supporting information

S1 File Corrected Admasu Thesis data. (SAV)

## Abbreviations

AIDS: Acquired Immune Deficiency Syndrome
AOR: Adjusted odd ratio
ART: Anti-Retroviral Therapy
AZT: Zidovudine
ATV: Atazanavir
CART: Combine Anti-Retroviral Therapy
CDC: Centre for Disease Control and Prevention
CD4: Cluster of Differentiation
CR1: Complement receptor type
DTG: Dolutegravir
EPHI: Ethiopia Public Health Institutes
HAART: Highly Active Antiretroviral Therapy
Hgb: Hemoglobin
HMIS: Health Management Information System
HTN: Hypertension
HIV: Human Immunodeficiency Virus
HSC: Hemantipoetic stem cells
IL-1β: Interleukin-1β
LPV: Lopinavir
MOH: Ministry of Health
NRTI: Nucleoside reverse transcriptase inhibitors
PI: Protease Inhibitors
PLT: Platelets
PUD: Peptic ulcer disease
R: Ritonavir
RBC: Red blood cells
SPSS: Statistical Package for Social Scien- ce
TB: Tuberculosis
TDF: Tenofovir
TNF: Tumor necrosis factor alpha
VL: Viral loads
UNAIDS: United Nations joint program on HIV/AIDS
WBC: white blood cells
WHO: World Health Organization
